# Petition of the Late Surgeon-General

**Published:** 1865-01

**Authors:** William A. Hammond

**Affiliations:** New York


### PETITION OF THE LATE SURGEON-GENERAL.

*To the Honorable the Senate of the United States:*

The undersigned, a resident of the City of New York, and late Surgeon-General of the Army of the United States, respectfully represents—

That he has been deprived of his Commission of Surgeon-General, and prohibited from again holding office under the Government of the United

States by the sentence of a General Court Martial, under circumstances which he prays your Honorable Body to inquire into before confirming the appointment which has been made of his successor.

He respectfully requests that your Honorable Body will cause to be printed and will examine the record of the Court Martial, and hear such additional evidence, he was, by the neglect of the Judge Advocate to summon his principal witness, and by other circumstances, unable to lay before the Court, but which it is now in his power to adduce.

He affirms, in all sincerity, that he believes the finding and sentence of the Court to be illegal, and not warranted by the facts of the case; that they were procured by conspiracy and false testimony, and that consequently his removal from office, and the disability placed upon him, are unjust and wrongful.

By the failure to prosecute the civil suit instituted against him by order of the Secretary of War, and by the fact that there is no appeal from the decision of a Court Martial to a higher Court, your petitioner is debarred all opportunity of vindicating his unjustly aspersed character, but such as your Honorable Body may see fit to afford him.

For these reasons, he prays your Honorable Body to inquire into all the circumstances connected with his recent trial and dismissal, and to suspend action in the matter of confirming the appointment of his successor, till such inquiry is made with the view that, should it appear that your petitioner has suffered injustice, he may be, by Act of Congress, restored to the position from which he has been displaced.

And your petitioner, as in duty bound, will ever pray, etc.

WILLIAM A. HAMMOND, M. D.

New York, Dec. 25, 1864.

